# Microglia involvement in sex-dependent behaviors and schizophrenia occurrence in offspring with maternal dexamethasone exposure

**DOI:** 10.1038/s41537-022-00280-6

**Published:** 2022-09-08

**Authors:** Chan Rim, Hyun-Sun Park, Min-Jung You, Bohyun Yang, Hui-Ju Kim, Soyoung Sung, Min-Soo Kwon

**Affiliations:** 1 Department of Pharmacology, Research Institute for Basic Medical Science, School of Medicine, CHA BIO COMPLEX, CHA University, Seongnam-si, Gyeonggi-do Republic of Korea; 2 Department of Biochemistry, College of Medicine, Inje University, Busan, Republic of Korea

**Keywords:** Molecular neuroscience, Schizophrenia

## Abstract

Fetal microglia that are particularly sensitive cells to the changes *in utero* environment might be involved in the sex-biased onset and vulnerability to psychiatric disorders. To address this issue, we administered a 50 µg/kg dexamethasone (DEX) to dams subcutaneously from gestational days 16 to 18 and a series of behavioral assessments were performed in the offspring. Prenatal exposure to dexamethasone (PN-DEX) induced schizophrenia (SCZ)-relevant behaviors in male mice and depressive-like behavior in female mice. SCZ-relevant behavioral patterns occurred in 10-week-old (10 W) male mice but not in 4-week-old (4 W) male mice. Microglia in the medial prefrontal cortex (mPFC) and the striatum (STR) of 10 W males prenatally treated with dexamethasone (10 W PN-DEX-M) showed hyper-ramified morphology and dramatically reduced spine density in mPFC. Immunofluorescence studies indicated that microglia in the mPFC of the 10 W PN-DEX-M group interacted with pre-synaptic Bassoon and post-synaptic density 95 (PSD95) puncta. PN-DEX-M also showed significantly changed dopamine system proteins. However, a testosterone surge during adolescence was not a trigger on SCZ-relevant behavior occurrence in 10 W PN-DEX-M. Furthermore, females prenatally treated with dexamethasone (PN-DEX-F) displayed depressive-like behavior, in addition to HPA-axis activation and inflammatory microglial phenotypes in their hippocampus (HPC). We propose that altered microglial function, such as increased synaptic pruning, may be involved in the occurrence of SCZ-relevant behavior in PN-DEX-M and sex-biased abnormal behavior in the PN-DEX model.

## Introduction

Schizophrenia (SCZ) is a serious mental illness characterized by psychotic symptoms, cognitive impairment, and functional decline in daily life. Reportedly, <1% of the global population suffer from SCZ, the onset of which occurs in early adulthood, following adolescence^1^. Several studies have suggested that SCZ is more common in men than in women^2^.

A growing body of research continues to explore the possibility that prenatal stress may act as a risk factor that increases the probability of SCZ onset during adulthood^3^. Several types of stressors, including social defeat, chronic stress, and dexamethasone (DEX), affect neurodevelopment as well as the hypothalamic-pituitary-adrenal axis (HPA axis) and epigenetic landscape, via increased maternal glucocorticoid (Gc) levels which are transmitted to the fetus through the placenta^4,5^. However, causal factors that initiate the onset of SCZ as well as mechanisms that underly the sculpting of the vulnerable central nervous system (CNS) by prenatal stress remain unclear.

Microglia are the primary innate immune cells of the CNS. They play an important role in neuronal maturation and homeostasis by establishing contact with neurons^6^. Microglia, which play a dual role by both eliminating excessive synapses and promoting synaptogenesis, act as primary organizers of synapses and neuronal circuits^7^. Extensive research into the role of microglia in synaptic development has indicated that dysfunctional microglia are associated with several neuropsychiatric disorders^8,9^. With respect to SCZ patients, in particular, excessive pruning of neuronal synapses by microglia results in low synaptic density^10^ and abnormal behavior^11,12^. In humans, synaptic density peaks during adolescence and gradually decreases as adulthood approaches^13^. Thus, excessive reduction of synaptic spine induced by biological and/or environmental factors during this process may induce SCZ^14^.

The dopamine theory in SCZ propounded by several studies postulates that the dysregulation of two dopaminergic pathways stemming from the ventral tegmental area, including mesolimbic (midbrain to striatum) and mesocortical (midbrain to the prefrontal cortex), are associated with schizophrenic symptoms^15,16^. These two dopamine circuits are reportedly vulnerable to stress^17^. The prefrontal cortex (PFC) is known as a stress-sensitive region of the brain^18^. A PFC that is subjected to excessive stress induces an imbalance in the dopamine system^19^. Dysregulation of D1R and D2R (dopamine receptor)-positive GABAergic medium spiny neurons in the striatum (STR) also contributes to the occurrence of SCZ^20^. Interestingly, the dopamine receptors, D1 and D2, localized in both pre-and post-synapses may be regulated via synaptic pruning, but not via synapse formation^21^.

Microglia also play a role in sex differentiation in the developing brain^22^. According to a previous study^23^, microglia show sex-biased morphological features and neuroinflammation. Additionally, sex-specific transcriptional profiles of microglia have been observed^24,25^. Thus, exploring the sex-dependent role of microglia may be key to understanding sex-biased psychiatric disorders and their underlying pathophysiologies.

The current study was conducted to elucidate possible mechanisms underlying prenatal stress -induced SCZ-relevant behaviors in mice. To mimic elevated stress hormone levels in vivo, we administered dexamethasone (DEX), a synthetic glucocorticoid, to female mice during late pregnancy. We found that prenatal DEX exposure (PN-DEX) evoked SCZ-relevant behavior in male offspring after adolescence while inducing depressive-like behavior in female offspring.

## Materials and methods

### Experimental animals

Thirty-six 13-week-old pregnant female C57BL/6 mice (Koatec Inc., Korea) and their pups used in all experiments were housed in cages holding 3–5 animals each, under specific pathogen free (SPF) conditions at 22 ± 0.5 °C and an alternating 12-h light–dark cycle at the CHA BIO COMPLEX animal facility (Seongnam, Korea), and supplied with food and water ad libitum. The animals were acclimatized to the laboratory for 1 week before being subjected to experiments. In order to reduce time-dependent variability, all experiments were performed during the light phase of the cycle. All experimental animals were handled in accordance with the guidelines of the Institutional Animal Care and Use Committee of CHA University (IACUC2100037) and ARRIVE reporting guidelines. We follow the “Sex and Gender Equity in Research-SAGER-guidelines”.

### Prenatal dexamethasone (DEX) treatment

The experimental model described by Nagano et al. was adopted with some modifications^26^. In brief, to mimic the induction of stress hormones during the prenatal period, a dose of 50 µg/kg dexamethasone (DEX, Sigma D1756, USA) or physiological saline was injected subcutaneously into pregnant mice once a day from gestational day (GD) 16 to 18. The dose of DEX needed was determined by taking the physiological glucocorticoid levels induced by stress conditions estimated by a previous study of ours^27^ into consideration. Two days after birth, pups were randomly assigned to each experimental group. After the weaning period, the pups were categorized into 3–5 animals per cage based on their sexes. To rule out to disturbed offsprings’ brain development by abnormal maternal care on pups after maternal dexamethasone injection, we checked that dam fed the breast milk to pups and made comfortable bedside with additional enrichment. We randomly grouped the offsprings that came from several mothers with same treatment for molecular experiments to exclude the selected bias.

### Behavioral tests

The mice were allowed to acclimatize to the testing room for at least 30 min before performing assessments. A previous study^26^, indicated that DEX may induce behavioral changes in the offspring of DEX-injected mice, 10 d after birth. Therefore, all behavioral assessments were conducted during the light cycle (between 9:00 a.m. and 7:00 p.m.) from 11 to 16 weeks after birth. The behavioral assessments were conducted in series as described in our previous studies^28^. The details of each behavioral test and animal number were described in the Supplementary Information.

### Microglia and synaptic protein colocalization

Bassoon and Post-synaptic density protein 95 (PSD95) puncta, as well as pre-and post-synaptic markers, which were colocalized with Iba-1 positive cells, respectively, were analyzed using Huygens professional software for Mac (Scientific Volume Imaging, Netherlands) and Image J (National Institutes of Health, Bethesda, MD, USA). Briefly, z-stacked confocal images (1 µm slices) were converted to maximum-intensity projection images using ImageJ software. In order to obtain clear and noise-reduced images, we conducted a deconvolution process using Huygens professional software. Next, a colocalization analysis tool with identical threshold settings was used throughout the study; 6–10 Iba-1 positive cells per mouse (3 mice per group) were analyzed.

### Microglial cell morphometrics

#### Skeletal and Sholl analysis

Microglial morphology (Leica TCS SP5 II confocal microscope) was analyzed according to a previously described method^29^. The size of microglia in the regions of interest (ROIs) was manually calculated using the freehand tool in ImageJ software. The number of microglial branch intersections, Endpoints/cells, and branch length/cells were automatically calculated using the Skeletal/Sholl analysis plugin. A total of 200 to 300 microglial cells per group (3 mice, 5–6 fields per group) were used for skeletal analysis. Fifteen single microglia per mouse (3 mice per group) were randomly selected and analyzed for Sholl analysis. The details of analysis were described in the Supplementary Materials.

### Golgi staining

To determine synaptic density, we performed Golgi staining using a FD Rapid GolgiStain^TM^ kit (FD NeuroTechnologies, Columbia, MD, USA) according to the manufacturer’s protocols. Dendritic spines were observed under a Zeiss Axioscan z1 microscope (Zeiss, Germany). Spine density and dendrite length were quantitatively analyzed by counting spines and dendrites using SynPAnal software^30^. The experimenters were blinded to the groups and 10 dendrites per mouse were selected for further analyses. Details for Golgi staining were described in the Supplementary Information.

### Statistical analysis

Data are presented as mean ± standard error of the mean (SEM). Significance of the differences between groups was assessed via unpaired Student’s *t* tests and two-way analysis of variance (ANOVA) followed by Tukey’s post hoc test, using GraphPad Prism version 7 for Mac (GraphPad, La Jolla, CA). Statistical significance was set at *p* < 0.05. Detailed statistical analysis related to two-way ANOVA was described in Supplementary Information.

## Results

### Maternal dexamethasone (DEX) exposure induces different, sex-dependent, abnormal behaviors in offspring

To investigate the effect of maternal stress hormone induction on fetal microglia in vivo, we administered a synthetic glucocorticoid (Dexamethasone; DEX) to dams. Considering that the gestation period of a mouse lasts between 19–21 d, and that ~15–21 d mark the end of the trimester, DEX was administered subcutaneously once a day for 3 d from gestational days (GDs) 16 to 18 to induce consistent exposure of all fetuses to DEX. Determination of the dose (50 µg/kg) to be administered was based on the findings of a previous study^26^ as well as the estimated physiological concentration of glucocorticoids under stressful situations^27^. Pups were separated according to sex at the end of the lactation period. The number of litters of each pregnant dam was counted after parturition, and no significant differences in litter size or sex-bias between the vehicle (Veh) and prenatal dexamethasone exposed (PN-DEX) groups were observed (Fig. S1a, b). In addition, there were no significant differences between the body weights of adult PN-DEX and Veh mice (Fig. S1c).

**Fig. 1:**
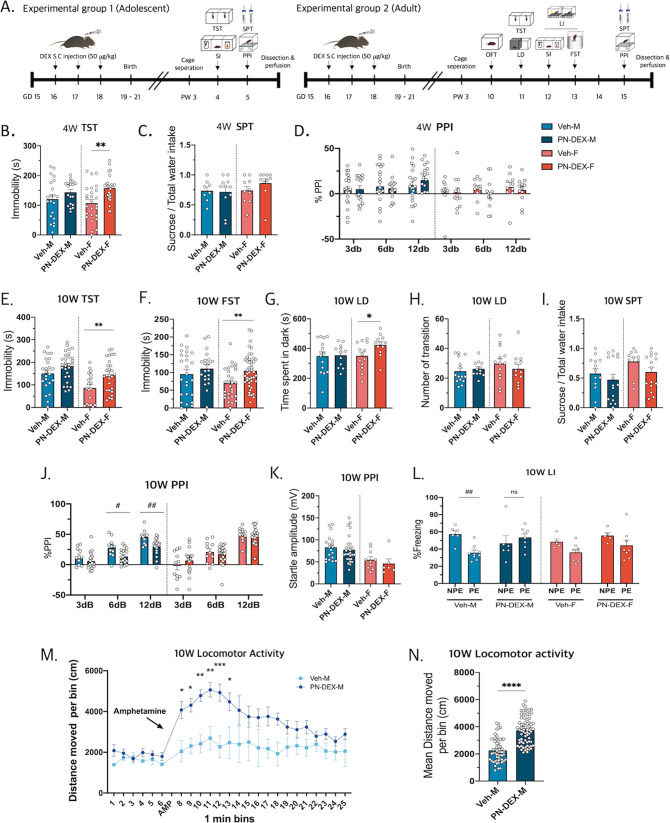
Maternal dexamethasone exposure induces sex-different behaviors in offspring. **A** Maternal dexamethasone exposure regimen: Dexamethasone (50 µg/kg) was subcutaneously injected into dams between gestational days 16 and 18, and a series of behavioral assessments were conducted on the offspring. **B** The 4 W PN-DEX-F showed increased immobility time in the TST. **C** No change in sucrose consumption was observed in any experimental group. **D** All experimental groups showed intact sensory gating functions at 3, 6, and 12 dB. **E** The 10 W PN-DEX-F showed increased immobility time in the TST and (**F**) FST. **G**, **H** 10 W PN-DEX-F showed increased dwelling time in the dark area in the LD test. **I** No significant changes were observed between sucrose consumption in the 10 W PN-DEX group and the 10 W Veh group. **J**, **K** 10 W PN-DEX-M showed significantly impaired sensory gating function in PPI but not in startle amplitude. **L** 10 W PN-DEX-M showed impaired LI. **M**, **N** Locomotor activity after amphetamine injection was increased. Data are presented as mean ± standard error of the mean (SEM). For the TST, FST, LD, SPT, and locomotor activity, an unpaired *t* test was conducted. *p < 0.05; **p < 0.01 compared with the Veh-M and Veh-F independently. For PPI and LI, two-way ANOVA followed by Tukey’s post-test was conducted; ^#^*p* < 0.05, ^##^*p* < 0.01.

Next, a series of behavioral assessments of PN-DEX offspring were conducted. The offspring were examined during their adolescent (4-week-old: 4 W) and adult (10-week-old: 10 W) stages (Fig. 1A). The 4 W PN-DEX-M did not exhibit depressive-like behavior and schizophrenia-relevant behavior, during the tail suspension test (TST), sucrose preference test (SPT), or prepurse inhibition (PPI) (Fig. 1B–D). However, results of the TST showed that the immobile time of 4 W PN-DEX-F was increased (Fig. 1B), although those of SPT, PPI, or social interaction (SI) did not show any significant changes (Figs. 1C, D, S1d, e). The results of both TST and FST indicated that the immobile time of 10 W PN-DEX-F was increased in a manner similar to that of 4 W PN-DEX-F, in addition, that of the light–dark test (LD) showed that time spent in the dark zone by 10 W PN-DEX-F was longer compared to that of 10 W Veh-F (Fig. 1E–H). The 10 W PN-DEX-F group did not show any significant changes in SPT, open field test (OFT), and SI (Figs. 1I, S1e–i). This suggested that exposure to PN-DEX induced depressive-like behaviors in the 4 W PN-DEX-F group, which behavior continued until the adult stages. Although the PN-DEX-F group showed depressive-like behavior, the PN-DEX-M group did not (Figs. 1E–I, S1f–i).

Interestingly, we found that, as opposed to the 4 W PN-DEX-M group, the 10 W PN-DEX-M group exhibited some major characteristics of the animal schizophrenia model, such as significant impairment of prepulse inhibition and latent inhibition (LI), whereas the 10 W PN-DEX-F group did not (Fig. 1J–L). Locomotor activity after being injected with the psychotropic drug, amphetamine (AMP), was measured to verify experimental results. The 10 W PN-DEX-M group showed increased locomotor activity following AMP treatment, compared with that of the Veh group (Fig. 1M, N).

### Change of microglia morphology and gene expression in male offspring with maternal dexamethasone exposure

First, we investigated factors that contribute to schizophrenia-relevant behavior in 10 W PN-DEX-M mice. At puberty, the number of dendritic spines in the gray matter of the human and non-human medial prefrontal cortex (mPFC) is decreased due to synaptic pruning^31^. Therefore, we hypothesized that schizophrenia-relevant behavior seen in 10 W PN-DEX-M may have been mediated by microglia.

**Fig. 2:**
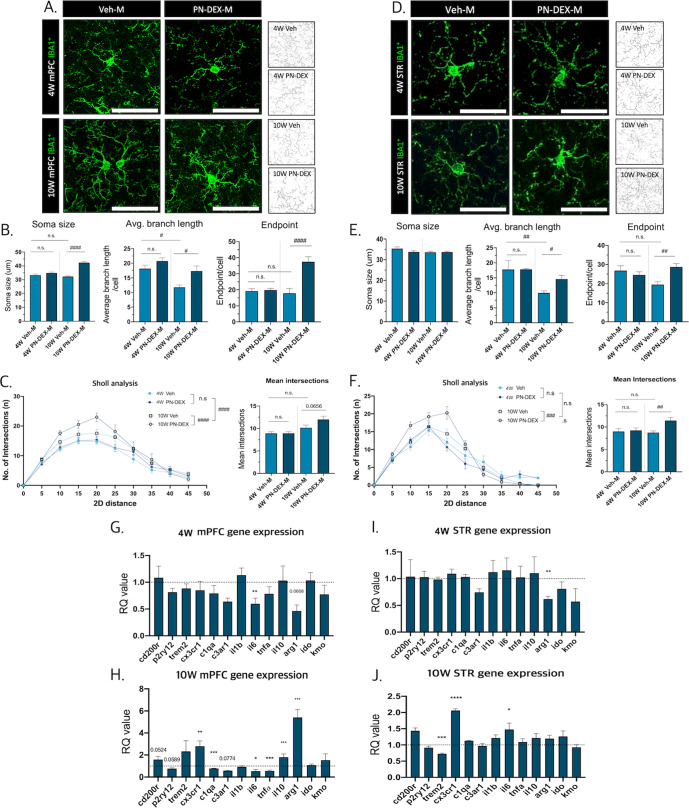
Maternal dexamethasone exposure induces microglial changes in the mPFC and the striatum of offspring. **A** Representative images of the morphology of Iba-1 positive cells in the medial prefrontal cortex (mPFC) according to time point and sex, (40× image, scale bar = 50 µm). **B** Soma size, branch length, and the number of endpoints in Iba-1 positive cells in the mPFC were calculated. **C** Sholl analysis indicated that mPFC microglia had a hyper-ramified morphology. **D** Representative images of the morphology of Iba-1 positive cells in the striatum (STR) according to time point and sex (40× image, scale bar = 50 µm). **E** Soma size, branch length, and the number of endpoints of Iba-1 positive cells in the STR were calculated. **F** Sholl analysis showed that striatal microglia had a hyper-ramified morphology. **G** qRT-PCR analysis to determine gene expression patterns in the mPFC of 4 W PN-DEX-M compared to that of 4 W Veh-M, and (**H**) the mPFC of 10 W PN-DEX-M compared to that of 10 W Veh-M. **I** 4 W PN-DEX-M STR, (**J**) 10 W PN-DEX-M STR. The RQ values are the ratios of the respective genes as a percentage of Veh. Data are presented as mean ± standard error of the mean (SEM). An unpaired *t* test was used for qRT-PCR; *p < 0.05; **p < 0.01; ***p < 0.001 compared with the Veh-M and Veh-F independently, For morphology analysis, two-way ANOVA followed by Tukey’s post-test was conducted; ^#^*p* < 0.05; ^##^*p* < 0.01; ^####^*p* < 0.0001.

We analyzed changes in the microglial morphology of 4 and 10 W PN-DEX-M groups. To precisely depict morphological changes in microglia, we selected two different but widely used methods, and modified the pipelines to suit our experimental environment. First of all, microglial numbers both in mPFC and striatum (STR) were not changed by maternal DEX exposure (Fig. S2a–d). According to the 2D skeletal analysis^32^ there was no significant change in endpoints, or average branch lengths in the mPFC of the 4 W PN-DEX-M group compared to that of the 4 W Veh-M group. By contrast, the mPFC of the 10 W PN-DEX-M showed enlarged microglial soma, increased endpoints, and average branch lengths (Fig. 2A, B). Interestingly, these changes were not found in the microglia of the 4 and 10 W PN-DEX-F groups (Figs. S3a–c, S4a–c). Secondly, to verify increased microglial ramification, we conducted a Sholl analysis, which assesses the complexity of cellular branches^33^. The Microglial Sholl analysis revealed that the number of intersections in the microglia of the 10 W PN-DEX-M group was increased (Fig. 2C). By contrast, there were no significant differences between either 4 W PN-DEX-F and Veh-F groups or between 10 W PN-DEX-F and Veh-F groups (Figs. S3d, S4d). These results indicated that the mPFC of the PN-DEX-M group had undergone morphological changes to a hyper-ramified state after adolescence, compared to that of the Veh-M group.

Next, we analyzed microglial signature genes and functional regulators involved in neuronal interactions in the mPFC. In particular, *Cx3cr1*, which is related to microglia-neuron contact and microglial migration, was greatly increased in the 10 W PN-DEX-M group compared to 10 W Veh-M. With respect to cytokines, *Il-1β* expression remained unchanged, but *Il-6* and *TNF-α* were significantly decreased in 10 W PN-DEX-M. On the other hand, anti-inflammatory genes, such as *Il-10*, and *Arg-1* were highly increased only in 10 W PN-DEX-M (Fig. 2H). Neither 4 W PN-DEX-M (Fig. 2G), nor 4 W or 10 W PN-DEX-F showed such changes (Figs. S3e, S4e).

We also analyzed microglial morphology and gene expression in the striatum, a region that is functionally related to the prefrontal cortex^34^. There were no significant changes between the morphologies of striatal microglia in the 4 W Veh-M and 4 W PN-DEX-M groups (Fig. 2D–F), or the 4 W PN-DEX-F group (Fig. S3f–i). Microglial signature genes and cytokine genes in the 4 W PN-DEX-M group remained unchanged, except for the mRNA expression of *Arg1* (Fig. 2I). However, morphology of the microglia in the striatum of the 10 W PN-DEX-M group changed to a hyper-ramified state similar to that of the microglia of the mPFC. Although the size of their soma did not increase, the endpoint and average branch length of the striatal microglia of the 10 W PN-DEX-M group increased, (Fig. 2D–F), as opposed to those of 10 W PN-DEX-F, which did not (Fig. S4f–i). In addition, the gene expression patterns in the striatum of 10 W PN-DEX-M were different from those in its mPFC. In the striatum of 10 W PN-DEX-M, *Trem2*, which is the regulator of the phagocytic function of microglia, was decreased, while *Cx3cr1* and *Il-6* were increased (Fig. 2J). Interestingly, there were no significant changes in gene expression in the striatum of 4 W PN-DEX-F mice, except for that of *Arg1* (Fig. S3j). On the other hand, expression of *C1qa* and *C3ar1*, which encode complement proteins and their receptors, respectively, as well as the expression of *Cx3cr1* were decreased in 10 W PN-DEX-F (Fig. S4j).

### Involvement of microglia on synaptic density and dopamine system in male offspring with maternal dexamethasone exposure

**Fig. 3:**
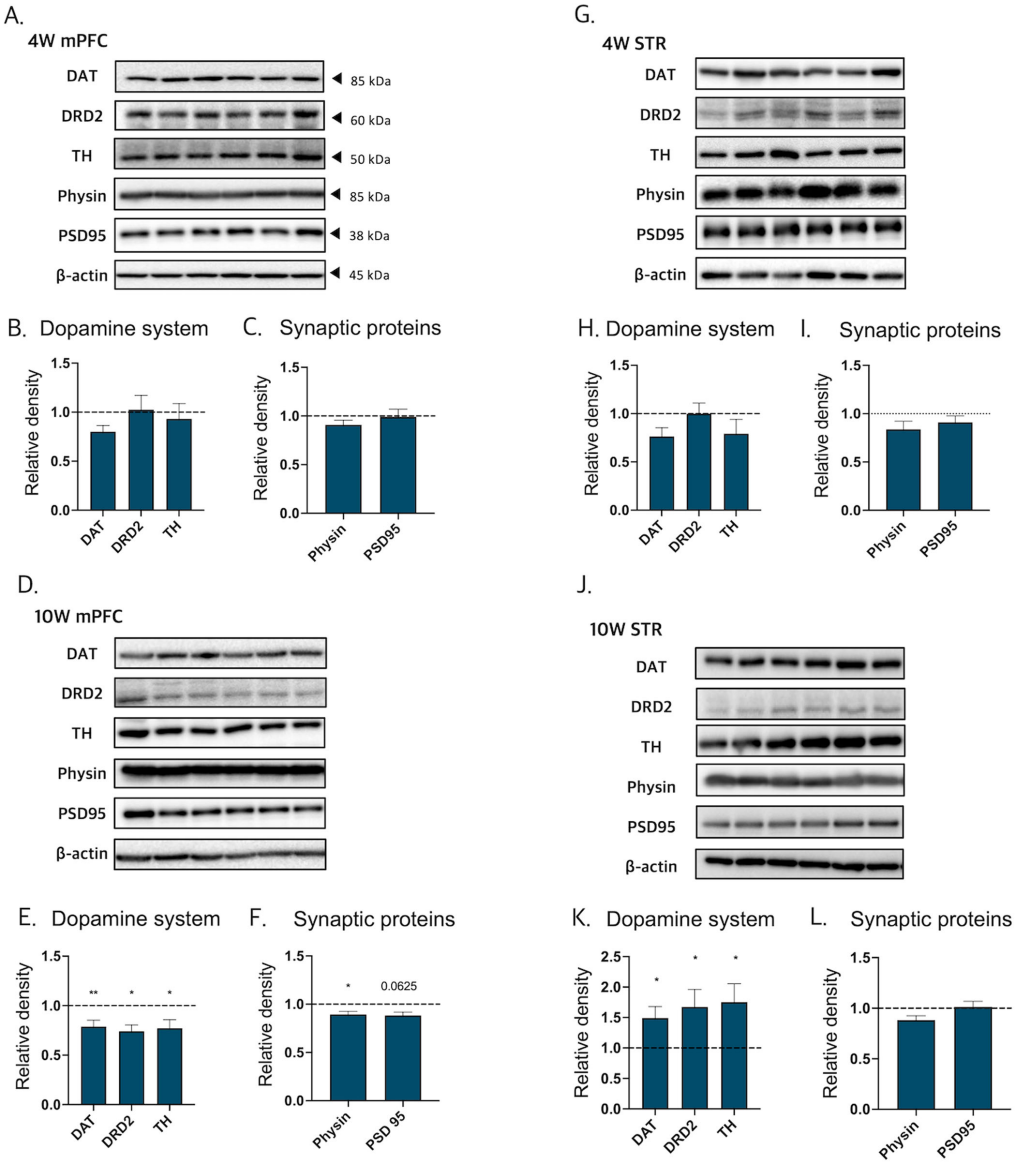
Change in synaptic protein and the dopamine system in male offspring with maternal dexamethasone exposure. Dopamine-related and synaptic protein levels were assessed via western blot analysis, and their expression was quantified using ImageJ. Relative density is defined as the ratio of respective protein density as a percentage of beta-actin density. **A** Representative bands of dopamine-related proteins and synaptic proteins in the 4 W mPFC. **B** Quantification of the relative density of dopamine-related proteins. **C** synaptic proteins in the 4 W mPFC. **D** representative band of dopamine-related protein and synaptic protein in the 10 W mPFC. **E** Quantification of the relative density of dopamine-related proteins and (**F**) synaptic proteins in the 10 W mPFC. **G** Representative bands of dopamine-related proteins and synaptic proteins in the 4 W STR. **H** Quantification of the relative density of dopamine-related proteins and (**I**) synaptic proteins in the 4 W STR. **J** Representative band of dopamine-related and synaptic proteins in the 10 W STR. **K** Quantification of the relative density of dopamine-related proteins and (**L**) synaptic proteins in the 10 W STR. The data are shown as mean ± standard error of the mean (SEM). For the purpose of statistical analyses, we conducted an unpaired *t* test; *p < 0.05, and **p < 0.01, compared with Veh-M.

**Fig. 4:**
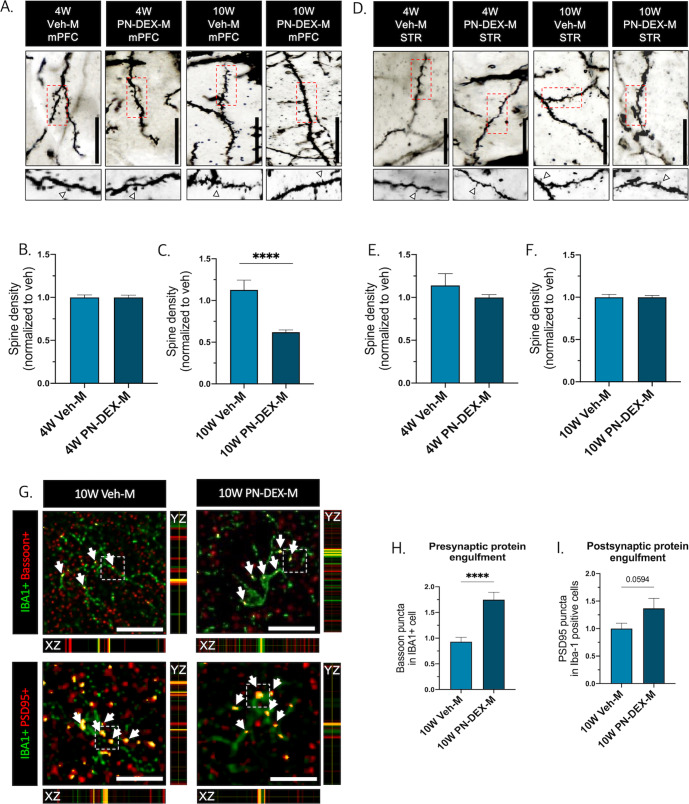
Increased microglia engulfment of pre-/post-synaptic proteins in the mPFC of 10 W PN-DEX-M. **A** Representative images of Golgi staining used to calculate spine density in 4 W and 10 W mPFC. **B** Spine density of 4 W PN-DEX-M mPFC, and (**C**) 10 W PN-DEX-M mPFC, normalized to the spine density of Veh-M. **D** Representative images of Golgi staining used to calculate spine density in 4 W and 10 W STR. **E** Spine density of 4 W PN-DEX-M STR, and (**F**) 10 W PN-DEX-M STR, normalized to the spine density of Veh-M. **G** Representative images of Bassoon (pre-synaptic marker) and PSD95 (post-synaptic) colocalized with Iba-1 positive cells in 10 W mPFC (40× image, scale bar = 25 µm). **H**, **I** Quantification of Bassoon and PSD95 puncta in Iba-1 positive cells of 10 W mPFC. Data are presented as mean ± standard error of the mean (SEM), For the purpose of statistical analysis, we conducted an unpaired *t* test; ***p < 0.001; ****p < 0.0001, compared with the Veh-M.

Dopamine signaling in the mPFC is associated with higher mental functions involving cognitive, emotional, and motivational processes^35,36^. In the mPFC of 10 W PN-DEX-M mice, the expression of factors that facilitate dopamine synthesis and transport, including dopamine receptor D2 (DRD2), dopamine transporter (DAT), and tyrosine hydroxylase (TH), were slightly decreased (Fig. 3D, E). In addition, synaptophysin and post-synaptic density protein 95 (PSD95) as well as pre-and post-synaptic proteins in 10 W PN-DEX-M were also slightly decreased (Fig. 3D, F). Conversely, although dopamine-related protein levels in the striatum of 10 W PN-DEX-M were significantly increased, synaptic protein levels were not (Fig. 3J–L). These data indicated that prenatal DEX exposure induced an imbalance in the cortico-striatal dopaminergic pathway as well as an aberrant synaptic protein expression pattern in 10 W PN-DEX-M. Interestingly, no significant changes were observed in the expression of synaptic and dopamine-related proteins in the mPFC or the striatum of 4 W PN-DEX-M (Fig. 3A–C, G–I). Furthermore, there were no significant changes in dopamine-related proteins and synaptic proteins in either region of 4 W and 10 W PN-DEX-F, except for a decrease in DAT expression observed in the mPFC of 4 W PN-DEX-F (Figs. S3k, l, S4k, l). A recent study reported that the microglial dopamine receptor (DR) is associated with synapse elimination^37^. Therefore, we hypothesized that microglial changes may lead to the dysregulation of synaptic pruning and the dopamine system, and thus performed rapid Golgi staining to visualize synaptic spines in the mPFC and striatum. No changes were observed in the spine densities of both the mPFC and the striatum of 4 W PN-DEX-M, compared to those of 4 W Veh-M (Fig. 4A, B, D, E). By contrast, spine density in the mPFC of 10 W PN-DEX-M, was dramatically reduced, while that in the striatum was not (Fig. 4A, C, D, F). Considered together, these findings indicated that prenatal DEX exposure affects spine density as well as the dopamine system in the mPFC and striatum of adult male offspring. With respect to morphological changes in the microglia of the 10 W PN-DEX-M group, we hypothesized that microglia may be responsible for the decrease seen in the synaptic density of the mPFC. In order to verify this, we calculated Bassoon (a pre-synaptic marker) and PSD95 (a post-synaptic marker) puncta colocalized with IBA1 positive cells (Fig. 4G). In line with our Golgi staining and western blot results, we found significantly increased pre-synaptic Bassoon and post-synaptic PSD95 puncta colocalized with IBA1 positive cells in the mPFC of the 10 W PN-DEX-M group, compared to those of the 10 W Veh-M group (Fig. 4H, I). These data indicated that maternal DEX exposure may affect microglia-mediated synaptic elimination between adolescence and adulthood.

### Testosterone surge in adolescence is not related to the occurrence of schizophrenia-relevant behaviors in male offspring with maternal DEX exposure

**Fig. 5:**
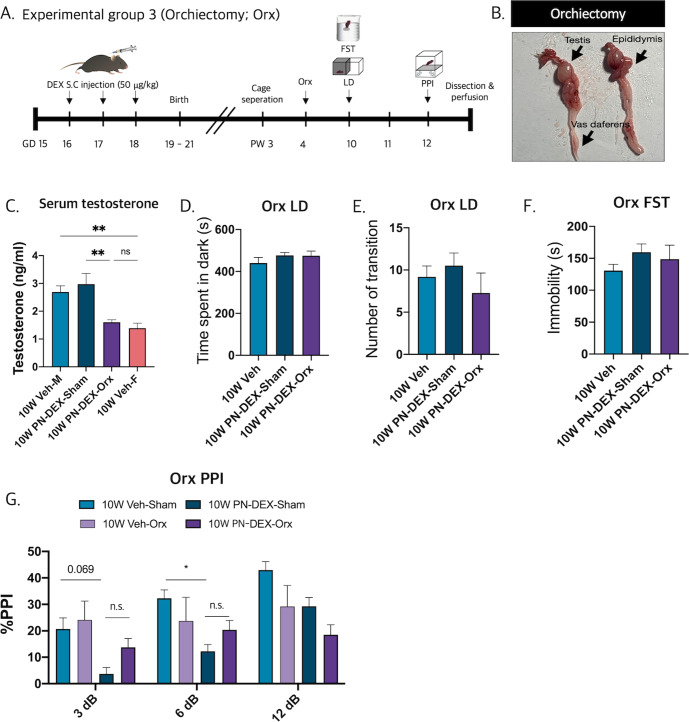
Testosterone surge in adolescent period is not related to the occurrence of schizophrenia-relevant behaviors with maternal dexamethasone exposure. **A** Experimental scheme for behavioral assessment following orchiectomy. **B** Representative images of dissected orchiectomy tissues. **C** Serum testosterone levels decrease after orchiectomy. **D**, **E** There were no significant behavioral changes in the LD, (**F**) FST, and (**G**) PPI groups. The data are expressed as the mean ± standard error of the mean (SEM), and one-way ANOVA followed by Tukey’s post-test was conducted; *p < 0.05; **p < 0.01.

The above results indicated that maternally DEX-exposed male offspring may experience the onset of SCZ-relevant behavior in early adulthood (10 W), after adolescence (4 W). Sex hormonal changes are the main events associated with adolescence, wherein testosterone acts as a determinant factor that produces maleness. Thus, we hypothesized that sex-biased biological events, such as testosterone surge may evoke SCZ-relevant behavior in PN-DEX-M, by inducing microglial changes after adolescence. To verify this speculation, we orchiectomized (Orx) 4 W PN-DEX-M to inhibit the testosterone surge and then performed PPI (Fig. 5A). The testes, epididymis, and the vas deferens were dissected (Fig. 5B). Orchiectomy decreased the serum testosterone levels in male mice to an extent which paralleled that of age-matched females (Fig. 5C). However, orchiectomy did not rescue impaired PPI in the PN-DEX-M group (Fig. 5F). By contrast, PN-DEX-Orx did not exhibit depressive-like behavior (Fig. 5C–E). In conclusion, the occurrence of SCZ-relevant behavior in the 10 W PN-DEX-M group was not due to the testosterone surge that occurs around puberty.

### PN-DEX-F showed HPA-axis activation and inflammatory microglia phenotype in the hippocampus (HPC)

**Fig. 6:**
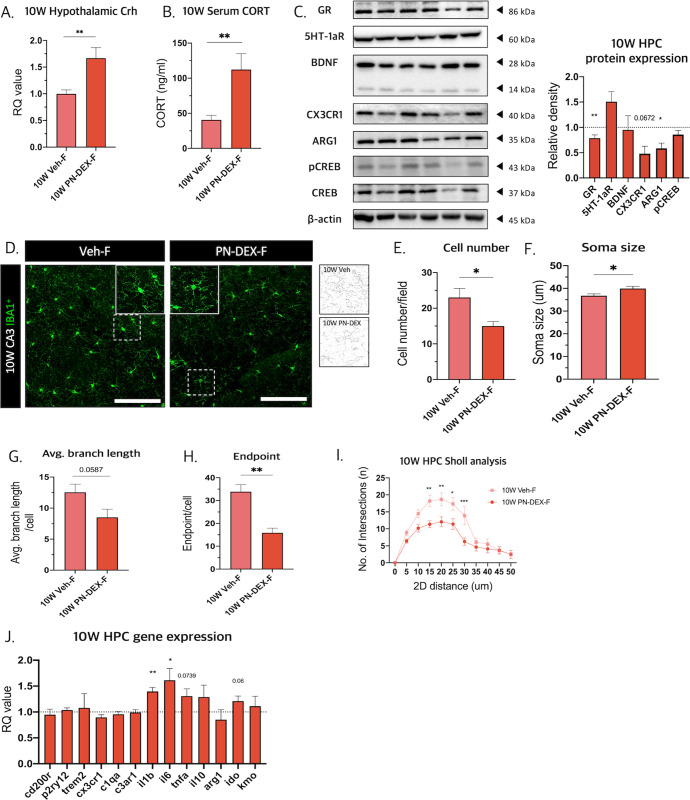
PN-DEX-F showed HPA-axis activation and inflammatory microglia phenotype in the hippocampus. **A** Hypothalamic Crh levels were measured using qRT-PCR. **B** Serum corticosterone levels were measured using ELISA. **C** Western blotting was used to assess several proteins related to depression-like behavior. Relative density is defined as the ratio of respective protein density as a percentage of β-actin density. **D** Representative images of Iba-1 positive cells in CA3 region of the hippocampus and skeletonized images (40× image, scale bar = 50 µm). **E** Cell number, (**F**) soma size, (**G**) branch length, and (**H**) number of endpoints in the HPC microglia (CA3 and dentate gyrus). **I** Sholl analysis showed that hippocampal microglia (CA3 and dentate gyrus) displayed de-ramified morphology. **J** qRT-PCR analysis used to determine gene expression patterns in 10 W PN-DEX-F HPC compared to 10 W Veh-F. Data are expressed as the mean ± standard error of the mean (SEM), and two-way ANOVA followed by Tukey’s post-test. Was conducted; ^#^*p* < 0.05; ^##^*p* < 0.01; ^###^*p* < 0.001. For the unpaired *t* test, *p < 0.05; **p < 0.01, compared with Veh-F.

To determine whether depressive-like behavior in PN-DEX-F is accompanied by molecular changes in the brain, we examined HPA axis-related factors, including hypothalamic corticotrophin-releasing hormone (CRH) and serum glucocorticoids (Gc). The 10 W PN-DEX-F group showed increased hypothalamic *Crh* levels and serum Gc levels compared to those of 10 W Veh-F (Fig. 6A, B), in accordance with which, hippocampal glucocorticoid receptor (GR) expression was decreased (Fig. 6C). This indicated that 10 W PN-DEX-F underwent HPA activation and GR resistance. Moreover, 10 W of PN-DEX-F showed a slight decrease in CX3CR1 and Arg1 protein expression (Fig. 6C).

Furthermore, we analyzed the morphology of HPC microglia. The number of microglia in the 10 W PN-DEX-F, group was decreased (Fig. 6D, E), in addition to which microglial somas were enlarged and displayed decreased branch arborization and branch lengths (Fig. 6F–H). The de-ramification of HPC microglia was confirmed by Sholl analysis (Fig. 6I). Moreover, the mRNA expression levels of the pro-inflammatory cytokines, Il-1β, *Il-6*, and *Tnf-*α, in the 10 W PN-DEX-F group were increased compared to those in 10 W Veh-F (Fig. 6J). Interestingly, 10 W PN-DEX-M showed no changes in microglial morphology, gene expression, protein expression, or the HPA axis (Fig. S5a–j). These data show that the microglia of 10 W PN-DEX-F exhibited an inflammatory microglial phenotype.

## Discussion

Maternal Gcs are transferred to the fetus through the placenta, and it has been reported that elevated maternal Gc levels are closely associated with the pathophysiology of several neuropsychiatric diseases in offspring^38^. In addition, neurodevelopmental changes, such as reduction of cortical thickness and changes in CNS cell populations were observed following exposure of the fetal brain to prenatal DEX^39,40^.

Abnormal developmental programming of fetal microglia by *in utero* stress situations has been indicated as a key mechanism underlying abnormal brain development^41^. These *in utero* stress to fetal microglia including maternal immune challenges and maternal stress hormone elevation alters microglial synaptic pruning and cytokine secretion and contributes to the prevalence of neuropsychiatric disorders such as major depressive disorder, and schizophrenia^42,43^.

Microglia also mediate several cellular functions, such as axon outgrowth, fasciculation, and cortical interneuron migration, during rodent fetal brain development between embryonic day 14 (E14) and 17.5 (E17.5)^44^. Because neurogenesis in most cortical and subcortical regions begins on or around E9.5^44,45^, we speculated that microglia-mediated synaptic formation and pruning that occurred during the perinatal period may have been induced by the DEX injection. Thus, exposure to DEX within the critical period of E15-E17, during maternity, may lead to a deterioration of brain development via microglial dysfunction. We also reported that DEX-treated fetal microglia displayed decreased phagocytic function, low proliferative capacity, and disrupted cytokine release, suggesting a dysfunctional state, such as cellular senescence, in vitro^46^. Therefore, DEX may disrupt normal microglial function, resulting in a state that is vulnerable to neuropsychiatric disorders.

Elevation of Gc-linked stress hormones during maternity may alter the cellular epigenetic landscape, including DNA methylation and histone acetylation in the CNS, thereby increasing its vulnerability to stress stimuli^4^. Exposure to stress during early life reportedly results in microglial DNA methylation in the STR and the HPC^47^. Glucocorticoid receptors (GRs) regulate a variety of genes via glucocorticoid response elements (GREs). Exposure to Gcs during fetal development induces epigenetic changes, including DNA methylation in GREs and other genes^48,49^. Cytoplasmic GRs interact with Gcs, resulting in the nuclear translocation of GRs and epigenetic modulation of the transcription of pro-/anti-inflammatory genes^50^. This indicates that stress-associated transcription, which is repressed by prenatal methylation of GRE, may be activated by demethylation of GRE via the second hit of Gc^51^. Additionally, FK506 binding protein 5, (FKBP5), which is an important modulator of the microglial stress response, regulates GR activity and its epigenetic modification by DEX^52^. A recent study revealed that epigenetic modification regulates microglial clearance in specific brain regions^53^. Considered together, these results indicate that exposure to DEX during maternity may lead to epigenetic modification of GRE-mediated transcription in the microglia of offspring, possibly accounting for the susceptibility to SCZ-relevant behavior in PN-DEX-M.

Perinatal androgen surge from the male testis and the subsequent local aromatization of testosterone to estradiol are crucial for the permanent modification of neuronal and microglial functions, as well as brain masculinization^54–56^. Testosterone surge during adolescence has been associated with several behavioral changes^57^. Progesterone, a testosterone precursor, antagonizes estradiol in synaptic remodeling, a process mediated by a progesterone receptor present on the rat microglia^58^. In addition, several sex hormones, including estradiol and luteinizing hormone, are related to the onset of SCZ^59^. Thus, we speculated that the occurrence of SCZ-relevant behavior during the 10 W adult stage, but not during 4 W adolescent stage, may have been evoked by a pubertal testosterone surge that arose in response to the second Gc hit. However, our hypothesis was contradicted by the observation that testosterone depletion by orchiectomy did not prevent the occurrence of SCZ-relevant behavior in PN-DEX-M. Nevertheless, the possibility that other factors may have induced the second Gc hit and excessive microglial synaptic pruning cannot be discarded. Thus, further studies aimed at determining the possibility of a second Gc hit are felt to be warranted.

In our study, microglia in the STR and the mPFC of PN-DEX-M showed hyper-ramified morphology characterized by enlarged soma and longer, more ramified branches at 10 W, but not at 4 W. These morphological changes, which allowed their processes to extend toward neuronal synapses and engulf synaptic compartments, may have enabled aberrant synaptic pruning in 10 W PN-DEX males. The association of microglia with synaptic elimination and the regulation of neuronal activity via complement cascades has been known for many years^60–62^. It has been reported that increased expression of the complement 4 protein (C4) in the synapses of patients with SCZ in the pathological state, may enhance microglial synaptic pruning via complement receptor 3 (CR3)^63^. A previous study observed an increase in neuronal C1q and microglial CR3 expression during the elimination of unnecessary synapses during developmental stages^64^. However, 10 W PN-DEX-M showed a reduction in *C1qa* and *C3ar1* mRNA expression. We speculate that active synaptic pruning of microglia by the complement system may have been evoked after 4 W, but before 10 W, resulting in a reduction of the spine density of 10 W PN-DEX-M. In addition to the complement system, other genes are involved in synaptic pruning. Increased *Cd200r* expression in microglia affects their interaction with neurons as well as their homeostatic state^65^. *Cx3cr1* and *P2ry12* signaling, which direct microglial branches toward synaptic spines, are also involved in microglial synaptic pruning^66^. The results of the present study revealed that increased expression of *Cd200r* and *Cx3cr1* in 10 W PN-DEX-M was accompanied by ramified microglial morphology. This suggests that microglia may remain in a ramified state that enables them to easily contact neurons following aberrant synaptic pruning during adolescence. Considered together, these results suggest that changes in various genes associated with synaptic pruning in microglia, such *as C1qa, C3ar1, Cx3cr1,* and *P2ry12*, may have affected a decrease in synaptic density as well as pre-/post- synaptic protein expression in 10 W PN-DEX-M. Despite several genes in the striatum of 4 W/10 W PN-DEX males and females being altered, synaptic density remained unchanged. Microglial synaptic pruning is a sensory experience-dependent^67^, as well as region- and time-dependent process^68^. This may explain the unchanged synaptic density seen in the STR. On the other hand, we found that the levels of DAT, TH, and DRD2 in the STR of 10 W PN-DEX males were significantly increased, whereas these proteins were decreased in the mPFC, suggesting the presence of hyperdopamine in the STR and hypo-dopamine in the mPFC. In summary, these data indicated that exposure to DEX during maternity induces SCZ-relevant behavior in male offspring via aberrant synaptic pruning and dysregulation of the dopamine system.

Depressive-like behavior observed in the 10 W PN-DEX-F group was accompanied by several changes in genes and proteins in the HPC. The expression levels of pro-inflammatory cytokine regulatory genes, including *Il-1β, Il6*, and *Tnf-α*, in the HPC of this group were increased, while those of ARG1 and CX3CR1 expression was slightly decreased. This suggests that prenatal DEX exposure induces inflammation in the HPC of female offspring via microglial activation. Similarly, hippocampal microglia show a pro-inflammatory phenotype and activated morphology, including enlarged soma as well as reduced branch length and arborization. Microglial activation may stimulate the HPA-axis, leading to increased hypothalamic *Crh* and serum Gc levels. Increased serum Gc levels caused by a chronically activated HPA axis may lead to GR insensitivity, which manifests in the form of decreased GR expression^69^. A decrease in ARG-1 positive microglia in the HPC is reportedly associated with depressive-like behavior^29^, while an imbalance in the hypothalamic-pituitary-adrenal axis (HPA axis) was also found to be related to depressive-like behavior^29,70^. These data provide mounting evidence supporting the previous contention that cellular and molecular mechanisms may underly depression-like behavior. Hippocampal GR resistance (decreased GR expression) activates microglia, via decreased expression of ARG1 and increased expression of pro-inflammatory genes, consequently aggravating HPA-axis imbalance (increased *Crh* and CORT) as well as depressive-like behavior^71^. Decreased hippocampal ARG1 expression, in particular, may be associated with enhanced stress vulnerability^72^.

A growing body of research indicates that early-life stress or prenatal stress may affect mouse and human behavior sex-dependently^73^. Biological gender has also been revealed to play a role in sex-dependent stress vulnerability and stress resilience^74^, brain sex differentiation, neuroimmunological function, and epigenetic modification^75^. Considering that developing microglia are closely associated with brain sex differentiation^76–78^ and stress responses^74^ during developmental stages, microglia may have acted as mediators of sex-specific behavior in the PN-DEX model. Several studies have suggested that microglia may display different functions^79^, transcriptomes^80^, and epigenetic landscapes depending on biological gender^55,75^. Although considered as being supported by controversial evidence, differences in phagocytic functions and cytokine production have been discerned between male and female microglia^23^.

In our prenatal DEX regimen, only 10 W PN-DEX-F showed HPA activation. HPA-axis activation which occurs exclusively in females may be associated with differences in the distribution of GR isoforms in the male placenta. In addition, synthetic Gcs and DEX evidently modulate GR expression only in female placentas^81^. The results of our study suggest that sex-differential distribution of GR isoforms in the placenta may explain the sex-biased behavior and HPA-axis programming evoked by the PN-DEX regimen. Therefore, we speculate that sex-dependent placental GR signaling and sex-dependent microglial development may have led to sex-dependent behavior in offspring following exposure to DEX during maternity.

The current study demonstrated that maternal DEX exposure induces sex-biased abnormal behavior, such as SCZ-relevant behavior in male offspring, and depressive-like behavior in female offspring. In addition, we propose that altered microglial functions, such as synaptic pruning, may be involved in the occurrence of SCZ-relevant behavior in PN-DEX-M although we did not examine microglia involvment on the maturation of dendritic spine. However, the following issues remain unresolved: (i) the nature of the association between the second Gc hit and microglial functional changes that led to SCZ-relevant behaviors seen only in 10 W PN-DEX-M; and (ii) the mechanism underlying sex-dependent differentiation of disease phenotypes in PN-DEX offspring. Thus, further studies using the PN-DEX model may be required to address these issues.

## Supplementary information

Supplementary materials

## Data Availability

All data generated or analysed during this study are included in the source data file and additional file.
