# Supplementary material for: Microglia involvement in sex-dependent behaviors and schizophrenia occurrence in offspring with maternal dexamethasone exposure
Source: Schizophrenia (Heidelb). 2022 Sep 8;8(1):71. doi: 10.1038/s41537-022-00280-6 (PMC9458670; doi:10.1038/s41537-022-00280-6)
Supplement: Supplementary file 1 — Supplementary materials [file 41537_2022_280_MOESM1_ESM.docx]

**Supplementary material**

**Material and Methods**

1. Behavioral tests

The mice were allowed to acclimatize to the testing room for at least 30 min before performing assessments. A previous study ^25^, indicated that DEX may induce behavioral changes in the offspring of DEX-injected mice, 10 d after birth. Therefore, all behavioral assessments were conducted during the light cycle (between 9:00 AM and 7:00 PM) from 11 to 16 weeks after birth. To assess anxiety/depressive-related behavior in mice, we conducted an open ﬁeld test (OFT), a light/dark exploration (LD), a tail suspension test (TST), a forced swim test (FST), and a sucrose preference test (SPT). Furthermore, in order to examine social behavior, we conducted a three-chamber social interaction (SI) test. Latent inhibition (LI), and prepulse inhibition (PPI) tests were performed to assess schizophrenia-related behavior. These behavioral assessments were conducted in series. Depending on the experimental design, the order of these behavioral assays were slightly changed. Each assessment was conducted during a single day, as described in our previous studies ^27^. Each behavioral test was described in the supplementary information. The observers were blinded to the groups, and the data so obtained were compared by two observers to minimize bias.

- 1. *The open field test*

Mice were placed in a 50×50 cm white Plexiglas box which was brightly lit using fluorescent room lighting; six 60 W incandescent bulbs were placed 4–6 feet above the box. Activity was recorded using a ceiling-mounted video camera and analyzed using EthoVision software (Noldus, Leesburg, VA). This software displays the paths taken by mice and measures the total distance moved and the number of entries into the center of the arena (central 17 cm square) during a 10 min session.

- 1. *Light dark exploration*

The apparatus used in this assessment was a box (30×30×30 cm) consisting of one brightly lit open chamber connected to a darkened, enclosed chamber. The chambers were connected by a small square hole (7 ×7 cm). Mice were placed in the corner of the lit chamber, facing away from the dark chamber, and the time spent in the dark chamber during a period of 10 min was manually measured.

- 1. *Tail suspension test*

The apparatus consisted of a cupboard with a hook attached to the top. Mice were suspended by securing the tail to the hook with adhesive tape which was wrapped around the tail. The tail was carefully suspended so as not to bend it, by keeping the tip of the tail 2 cm away from the top of the hook. Data pertaining to mice that climbed their tails were excluded. The time spent immobile during the 7 min testing period was measured by three observers who were blinded to the groups.

- 1. *Forced swim test*

Each mouse was placed in a 2 L Pyrex beaker (13 cm diameter, 24 cm height), which was filled with 24°C water to a depth of 17 cm, and forced to swim for 6 min. The duration of immobility was measured during the final 5 min of the test. Immobility was defined as the time each mouse spent floating without struggling and only making movements that were necessary to keep its head above water level. The time spent immobile during the 5 min testing period was measured by three blinded observers.

- 1. *Three-chamber social interaction*

The social interaction box was a rectangular, three-chambered box made of white Plexiglas (60 cm × 40 cm × 40 cm). Each chamber measured 20 cm × 40 cm × 40 cm. Dividing walls were also made of clear Plexiglas with a small opening (square, 6 cm × 6 cm) that allowed access to each chamber. Each test mouse was placed in the middle chamber and allowed to explore for 5 min. The doorways in the two side chambers were blocked with plastic boxes during the habituation phase. After 5 min of habituation, an unfamiliar mouse of the same strain, that had no prior contact with the subject mouse, was placed in one side chamber. The location of the stranger mouse was systematically alternated between the left- and right-chambers during the trial. The stranger mouse was enclosed in a cylinder made of transparent Plexiglas, which allowed nose contact through the holes, but prevented fighting. The cylinder was 15 cm in height, with a bottom diameter of 8 cm and holes spaced 1 cm apart. The cover, made of Plexiglas, was placed on top of the cylinder to prevent the test mouse from climbing ,also prevent the stranger mouse from escaping. Both doors to the side chambers were then unblocked, and the test mouse was allowed to explore the entire social interaction box for a 10-min session to measure sociality. Activity in the social interaction chamber was recorded, and the time spent in each chamber was measured using an EthoVision XT9 video tracking system. After the first 10 min session ended, each mouse was tested in a second 10-min session to check social preference for a new stranger, using a second unfamiliar mouse, which was also enclosed in a cylinder made of transparent Plexiglas, and placed in the chamber that had been empty during the first 10-min session. The test mouse had a choice between the first, already-investigated, unfamiliar mouse (stranger 1) and the second, unfamiliar mouse (stranger 2). As described above, the activity in the social interaction chamber was recorded, and the time spent in each chamber was measured using an EthoVision XT9 video tracking system.

- 1. *Latent inhibition*

This protocol was modeled after a previous study [1]. Each mouse group (control or amphetamine) was randomly subdivided into two groups: pre-exposed (PE); and non-pre-exposed (NPE). The mice were placed in a shuttle box (Jungdo Biotech, Seoul, Korea) with a speaker mounted on the back wall. PE mice were exposed to 40 tones (1 tone; 2000 HZ, 30 s duration) separated by 30 ± 40 s to randomize the inter-tone interval. The NPE mice were placed in the same enclosure for an equivalent amount of time. Immediately following pre-exposure, all mice were given three pairing trials of the 30 s tone, followed immediately by a 1 s, 0.3 mA foot-shock delivered through the floor. Pairing trials were separated by 180 s. Mice were returned to the enclosure the next day and presented with an 8 min tone presentation following a 180 s acclimation period. Freezing during tone presentation was measured using EthoVision XT9. The percentage of time spent freezing during tone presentation was measured, with LI being defined as the difference between the amounts of freezing in response to the tone by PE mice and NPE mice. The NPE groups were merged to obtain greater statistical power.

- 1. *Acoustic startle/Prepulse inhibition*

PPI testing, which adopted the method described by Dulawa et al.,(2000) with modifications, was performed in SR-LAB startle chambers (San Diego Instruments, San Diego, CA). Mice were exposed to 5 different types of discrete stimuli or “trials” as follows: a 40-msec broadband 120 dB burst (Pulse Alone trial); 3 different Prepulse + Pulse trials in which either 20-msec long 3 dB, 6 dB or 12 dB above background stimuli preceded the 120 dB pulse by 100 msec (onset to onset); and a No Stimulus trial, during which only background noise was presented. The trials were conducted in a pseudo-random order, separated by an average of 15 s (range,7–23 s). The test session began with a 5-min acclimation period, followed by four consecutive blocks of test trials. Blocks 1 and 4 consisted of six consecutive Pulse Alone trials, while blocks 2 and 3 each contained six Pulse Alone trials, five of each type of Prepulse + Pulse trials, and five No Stimulus trials. Thus, the entire 22-min session consisted of 62 test trials. PPI was calculated as follows: [100 – (Prepulse-Pulse trial/Pulse alone) × 100]. The pulse-alone trials in this calculation involved the averaged pulse-alone values for blocks 2 and 3.

1. Molecular works
   1. Immunofluorescence

For perfusion purposes, the mice were sacriﬁced following behavioral assessment. The mice were deeply anesthetized using sodium pentobarbital (100 mg/kg, i.p.) and perfused intracardially with heparinized saline, followed by ice-cold phosphate-buffered 4% paraformaldehyde (pH 7.4). Each brain was dissected and post-ﬁxed in the same ﬁxative at 4 °C. Next, brain blocks were cryoprotected in 30% sucrose for 24 h at 4 °C, following which 25 mm thick sections were obtained using an electronic cryotome (Leica CM 3050S, Germany). Sections were ﬁrst rinsed thrice (10 min each time) with 1% bovine serum albumin (BSA) in 0.1 M phosphate buffered saline (PBS). For permeabilization and blocking purposes, the tissues were pre-incubated in 0.1 M PBS containing 10% Bovine serum albumin (BSA; Roche, 10735086001), 3% Fetal bovine serum (FBS; Gibco, USA, 16000-044), 5% Normal donkey serum (NDS), and 0.3% Triton X-100 for 1 h at room temperature (RT). After rinsing twice (10–15 min each time) with 0.1 M PBS containing 0.5% BSA, sections were incubated with polyclonal anti-rabbit anti-Iba-1 antibodies (1:500; Wako, Japan # 019-19741), goat anti-Iba-1 antibodies (1:500; Novus, USA, #NB100-1028), mouse anti-Bassoon antibodies (1:1000; Novus, #NB120-13249), and rabbit anti-PSD95 (1:500; Abcam, UK, #ab238135) diluted with 0.1 M PBS containing 0.5% BSA at RT. Following overnight incubation, sections were rinsed and incubated with Alexa-488 conjugated anti-rabbit IgG antibodies (Invitrogen, USA, A21206), Alexa-488 conjugated anti-goat IgG antibodies (Invitrogen, A11055), Alexa-594 conjugated anti-rabbit IgG antibodies (Invitrogen, A21207), and Alexa-647 conjugated anti-mouse IgG antibodies (Invitrogen, A31571) in a 1:200 dilution with 0.1 M PBS containing 1% BSA for 1 h at RT. After rinsing with 0.1M PBS, coverslips were mounted using Prolong^TM^ Gold antifade mounting solution with DAPI (Invitrogen, P36931).

- 1. Western blot

Proteins in the medial prefrontal cortex (mPFC), striatum (STR), and the hippocampus (HPC) were extracted, and their expression levels assessed using western blotting. After dissecting, the mPFC, STR, and HPC, tissues were lysed with RIPA buffer (Sigma #R0278) and homogenized. To obtain pure proteins, debris was removed by ultracentrifugation following whole tissue homogenization. Protein concentrations were determined using a detergent-compatible protein assay reagent (Bio-Rad Laboratories, USA) with BSA as the standard. After adding dithiothreitol (5 mM) and bromophenol blue (0.1% w/v), the proteins were boiled, separated by electrophoresis on 10–15% polyacrylamide gels (Invitrogen), and transferred onto polyvinylidene difluoride (PVDF) membranes (Bio-Rad Laboratories). The membranes were blocked on a shaker for 1 h at RT with Tris-buffered saline/0.1% Tween-20 (TBS-T) and 5% nonfat dry milk. Primary antibodies were dissolved in the blocking buffer and the membranes were immunoblotted with antibodies against dopamine receptor D2 (DRD2; 1:200, Bioss #bs-1008R), dopamine transporter (DAT; 1:200, Abcam #ab111468), tyrosine hydroxylase (1:200, Abcam #ab112), post-synaptic density 95 (PSD95; 1:500, Abcam #ab18258), synaptophysin (Physin; 1:5000, Abcam, #ab14692), glucocorticoid receptor (GR; 1:1000, Santa Cruz Biotechnology, USA, #SC1004), 5-HT1a receptor (5-HT1aR; 1:1000, GeneTex, USA, # GTX104703), brain-derived neurotrophic factor (BDNF; 1:1000, Abcam #ab108319), CX3C chemokine receptor1 (CX3CR1; 1:1000, Abcam #ab8021), Arginase 1 (ARG1; 1:2000, Novus #NB100-59740), cAMP response element-binding protein (CREB; 1:500, Cell Signaling Technology # 9197S), hosphor-CREB (pCREB; 1:500, Cell Signaling Technology, USA, #9198S) and beta-actin (1:5000, Cell Signaling #4970S). The membranes were incubated in )oat anti-rabbit (1:10000, Enzo #ABI-SAB-300-J) or goat anti-mouse (1:10000, BETHYL, A120-101P) antibodies and dissolved in blocking buffer at room temperature for 1 h. The membranes were visualized using an ECL-plus solution (GE Healthcare, USA, RPN2106V1 and V2), following which they were exposed to chemiluminescence (LAS-4000; Fujifilm, Japan) to detect light emission. Western blot results were quantified using ImageJ 1.51 software (National Institutes of Health, Bethesda, MD, USA) following densitometric scanning of films.

- 1. Enzyme-linked immunosorbent assay (ELISA)

Mice were sacrificed, following which whole blood was collected via cardiac puncture, and serum was isolated and stored at − 80°C until assayed. Serum glucocorticoid and testosterone levels were determined using a corticosterone ELISA kit (Enzo, #ADI-900-097) and a testosterone ELISA kit (Enzo #ADI-900-065), respectively, according to the manufacturer’s instructions.

- 1. Quantitative Real-time Polymerase Chain Recation (qRT-PCR)

Following behavioral assessments, the mPFC, STR, and HPC were dissected to analyze mRNA expression. To extract RNA, frozen tissue was homogenized in 1 mL of TRIzol reagent per 100 mg of tissue (Life Technologies, 15596018). Chloroform was added to separate phase-containing RNA, while isopropyl alcohol was added to precipitate RNA. Each precipitated RNA pellet was air-dried and dissolved in DEPC-treated water (Life Technologies; AM9906). RNA concentration was determined by measuring absorption at 260 nm. Messenger RNA (mRNA) was reverse-transcribed into cDNA in 20 μL of reaction mix using a RevertAid First Strand cDNA Synthesis Kit (ThermoFisher Scientific, K1622). Quantitative PCR was performed using Power SYBR® Green PCR Master Mix (Life Technologies, 4367659). Primer sequences are listed (Table S1). Cyclic conditions consisted of initial enzyme activation at 95°C for 5 min, followed by 50 cycles of denaturation at 95°C for 20 s, annealing, and extension, including detection of SYBR Green bound to PCR product at 56°C for 40 s. Glyceraldehyde 3-phosphate dehydrogenase (GAPDH) was used as an internal control for normalization. Relative quantities of PCR fragments were calculated using the comparative CT method.

1. Microglial cell morphometrics
   1. Skeletal analysis
      Z-stacked confocal images (1 µm slices) were converted to maximum intensity projection images using ImageJ. To exclusively visualize positive staining, an unsharp mask filter was applied to stacked images and brightness and contrast were adjusted. Next, RGB images were converted to grayscale images, and a threshold was applied. To obtain clear and precise images, noise reduction techniques, such as removing outliers and de-speckling, were applied to binary images, followed by skeletonizing branches. Lastly, the analyze skeleton plugin was run and data were trimmed to remove skeleton fragments using Microsoft Excel.
   2. Sholl analysis

For the Sholl analysis, Single microglia in the 8-bit and z-stacked images were isolated by removing surrounding artifacts and other cell fragments via the eraser tool, which was originally built into ImageJ. The experimenter then drew a straight line from the center of the microglial soma to its longest branches using the line segment tool. Finally, using the Sholl analysis plugin, the first shell was defined as 10 µm outside the cell body, and each step was set to 5 µm. The number of intersections of microglial branches were automatically calculated Sholl analysis plugin.

1. Golgi staining

Mice were euthanized and their brains perfused with heparinized physiological saline. The brains were then immersed in solutions A and B for the impregnation step and stored at room temperature for up to 2 weeks in the dark. Next, the brains were transferred to solution C, following which 4 were stored in the dark for up to one week. Each brain was serially sectioned at a thickness of 100 µm using a cryotome (Leica Biosystems, Germany). The sections were mounted on gelatin-coated slides and allowed to dry naturally at room temperature. Mounted sections were rinsed with double-distilled water and allowed to react with an equal volume of solutions D and E in the dark for 10 min. Next, sections were rinsed in double-distilled water and dehydrated via an ascending alcohol gradient (50, 75, 80, 95, and 100%). After dehydration, the sections were cleaned in Histoclear (National Diagnostics, Georgia, USA) and cover-slipped with Permount solution (Fisher Chemicals #SP15-500 HS200).

**Supplementary table**

**Supplementary Table 1. Primer sequence**

Mouse

|  | Forward (5’-3’) | Reverse (5’-3’) |
| --- | --- | --- |
| GAPDH | CGACCTTCACCATTTTGTCTACA | GCTTAAGAGACAGCCGCATCT |
| Cd200r | AGGAGGATGAAATGCAGCCTTA | TGCCTCCACCTTAGTCACAGTATC |
| Trem2 | TGGGACCTCTCCACCAGTT | GTGGTGTTGAGGGCTTGG |
| Cx3cr1 | TGGCCCAGCAAGCATAG | CATGTCTGCTACCCTCACAAA |
| Il1b | GGCTGGACTGTTTCTAATGC | ATGGTTTCTTGTGACCCTGA |
| Il6 | GCAAGTGCATCATCGTTGTTCATAC | CCACTTCACAAGTCGGAGGCTTA |
| Tnfa | GAGTCCGGGCAGGTCTACTTT | CAGGTCACTGTCCCAGCATCT |
| Il10 | GCAGCTCTAGGAGCATGTGG | ACAGCCGGGAAGACAATAACT |
| Arg1 | GGAATCTGCATGGGCAACCTGTGT | AGGGTCTACGTCTCGCAAGCCA |
| Ido | GGCTTCTTCCTCGTCTCTCTATTG | TGACGCTCTACTGCACTGGATAC |
| Kmo | CCTGTAGAGGACAATATAGGATCAACAA | GCAAGCCCCATCTACTGCAT |
| C1qa | Qiagen PPM24525E | |
| C3ar1 | Qiagen PPM04821A | |

**Supplementary Table 2. Statistics of two-way ANOVA in behavior assessment in 4W/10W and Orx group.**

| **4W Behavior - PPI** | | | | | |
| --- | --- | --- | --- | --- | --- |
|  | **SS** (Sum-of-squares) | **Df**  (Degrees of freedom) | **MS**  (Mean squares) | **F**  (F ratio) | **p-value** |
| Treatment (Veh vs. PN-DEX) | 53.89 | 1 | 53.89 | 0.1386 | P=0.7104 |
| Decibel (3 dB, 6 dB, 12 dB) | 1002 | 2 | 500.8 | 1.288 | P=0.2799 |
| Interaction  Treatment x Decibel | 3476 | 6 | 579.3 | 2.273 | P=0.0388 |

| **10W Behavior - PPI** | | | | | |
| --- | --- | --- | --- | --- | --- |
|  | **SS** (Sum-of-squares) | **Df**  (Degrees of freedom) | **MS**  (Mean squares) | **F**  (F ratio) | **p-value** |
| Treatment * (Veh vs. PN-DEX) | 1417 | 1 | 1417 | 5.898 | P=0.0162 |
| Decibel **** (3 dB, 6 dB, 12 dB) | 41236 | 5 | 8247 | 34.33 | P<0.0001 |
| Interaction *  Treatment x Decibel | 2815 | 5 | 563 | 2.343 | P=0.0436 |
| **10W Behavior - LI** | | | | | |
| Treatment  (Veh vs. PN-DEX) | 89.23 | 1 | 89.23 | 0.5127 | P=0.4804 |
| Conditioning (NPE vs. PE) | 393 | 1 | 393 | 2.258 | P=0.1449 |
| Interaction **  Treatment x Conditioning | 1448 | 1 | 1448 | 8.319 | P=0.0078 |
| **10W Behavior – Locomotor activity** | | | | | |
| Treatment *  (Veh vs. PN-DEX) | 90715093 | 1 | 90715093 | 6.591 | P=0.0200 |
| Time **** (1 min. bins) | 132185617 | 23 | 5747201 | 16.79 | P<0.0001 |
| Interaction ****  Treatment x Time | 27350861 | 23 | 1189168 | 3.475 | P<0.0001 |

| **Orx Behavior - PPI** | | | | | |
| --- | --- | --- | --- | --- | --- |
|  | **SS** (Sum-of-squares) | **Df**  (Degrees of freedom) | **MS**  (Mean squares) | **F**  (F ratio) | **p-value** |
| Treatment **** | 7954 | 3 | 2651 | 10.40 | P<0.0001 |
| Decibel **** | 5763 | 2 | 2882 | 11.31 | P<0.0001 |
| Interaction *  Treatment x Decibel | 311.2 | 2 | 155.6 | 0.4002 | P=0.6712 |

**Supplementary Table 3. Statistics of two-way ANOVA in microglia morphology analysis.**

| **mPFC – Soma size** | | | | | |
| --- | --- | --- | --- | --- | --- |
|  | **SS** (Sum-of-squares) | **Df**  (Degrees of freedom) | **MS**  (Mean squares) | **F**  (F ratio) | **p-value** |
| Treatment  (Veh vs. PN-DEX) | 6210 | 1 | 6210 | 74.66 | P<0.0001 |
| Timepoint  (4W vs. 10W) | 1904 | 1 | 1904 | 22.89 | P<0.0001 |
| Interaction  Treatment x Timepoint | 3269 | 1 | 3269 | 39.3 | P<0.0001 |
| **mPFC – Endpoint** | | | | | |
| Treatment  (Veh vs. PN-DEX) | 580.1 | 1 | 580.1 | 14 | P=0.0013 |
| Timepoint  (4W vs. 10W) | 377.3 | 1 | 377.3 | 9.107 | P=0.0068 |
| Interaction **  Treatment x Timepoint | 506.7 | 1 | 506.7 | 12.23 | P=0.0023 |
| **mPFC – Average branch length** | | | | | |
| Treatment  (Veh vs. PN-DEX) | 94.09 | 1 | 94.09 | 8.922 | P=0.0073 |
| Timepoint *** (4W vs. 10W) | 137.4 | 1 | 137.4 | 13.02 | P=0.0018 |
| Interaction  Treatment x Timepoint | 12.48 | 1 | 12.48 | 1.183 | P=0.2896 |
| **mPFC – Sholl analysis** | | | | | |
| Treatment **** (Veh vs. PN-DEX) | 1117 | 3 | 372.3 | 20.04 | P<0.0001 |
| Timepoint **** (4W vs. 10W) | 20714 | 9 | 2302 | 123.9 | P<0.0001 |
| Interaction ***  Treatment x Timepoint | 1039 | 27 | 38.47 | 2.071 | P=0.0013 |
| **mPFC – Mean intersections** | | | | | |
| Treatment  (Veh vs. PN-DEX) | 27.45 | 1 | 27.45 | 0.5374 | P=0.4641 |
| Timepoint  (4W vs. 10W) | 146.2 | 1 | 146.2 | 2.863 | P=0.0917 |
| Interaction *  Treatment x Timepoint | 27.45 | 1 | 27.45 | 0.5374 | P=0.4641 |

| **STR – Soma size** | | | | | |
| --- | --- | --- | --- | --- | --- |
|  | **SS** (Sum-of-squares) | **Df**  (Degrees of freedom) | **MS**  (Mean squares) | **F**  (F ratio) | **p-value** |
| Treatment  (Veh vs. PN-DEX) | 105.2 | 1 | 105.2 | 1.651 | P=0.1331 |
| Timepoint  (4W vs. 10W) | 151.6 | 1 | 151.6 | 2.379 | P=0.1233 |
| Interaction  Treatment x Timepoint | 144 | 1 | 144 | 2.26 | P=0.1991 |
| **STR – Endpoint** | | | | | |
| Treatment  (Veh vs. PN-DEX) | 69.39 | 1 | 69.39 | 3.553 | P=0.0741 |
| Timepoint  (4W vs. 10W) | 13.78 | 1 | 13.78 | 0.7056 | P=0.4109 |
| Interaction **  Treatment x Timepoint | 188.1 | 1 | 188.1 | 9.632 | P=0.0056 |
| **STR – Average branch length** | | | | | |
| Treatment  (Veh vs. PN-DEX) | 30.57 | 1 | 30.57 | 3.257 | P=0.0862 |
| Timepoint *** (4W vs. 10W) | 174.7 | 1 | 174.7 | 18.62 | P=0.0003 |
| Interaction  Treatment x Timepoint | 29.38 | 1 | 29.38 | 3.13 | P=0.0921 |
| **STR – Sholl analysis** | | | | | |
| Treatment **** (Veh vs. PN-DEX) | 436.2 | 3 | 145.4 | 10.29 | P<0.0001 |
| Timepoint **** (4W vs. 10W) | 10415 | 7 | 1488 | 105.3 | P<0.0001 |
| Interaction ***  Treatment x Timepoint | 717.7 | 21 | 34.18 | 2.418 | P=0.0006 |
| **STR – Mean intersections** | | | | | |
| Treatment  (Veh vs. PN-DEX) | 11.35 | 1 | 11.35 | 2.53 | P=0.1186 |
| Timepoint  (4W vs. 10W) | 17.96 | 1 | 17.96 | 4.002 | P=0.0514 |
| Interaction *  Treatment x Timepoint | 26.03 | 1 | 26.03 | 5.802 | P=0.0201 |

**Supplementary Table 4. The number of mice used in behavior assessment.**


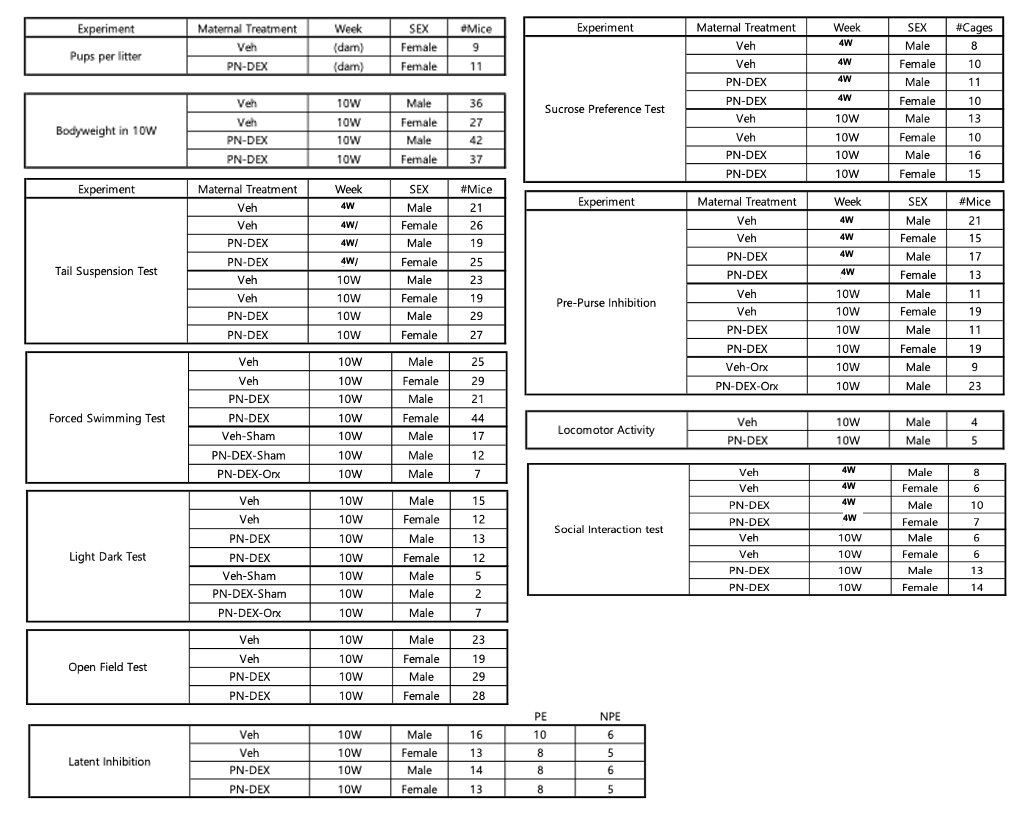


**Supplementary Table 5. The number of mice used in molecular works.**

**
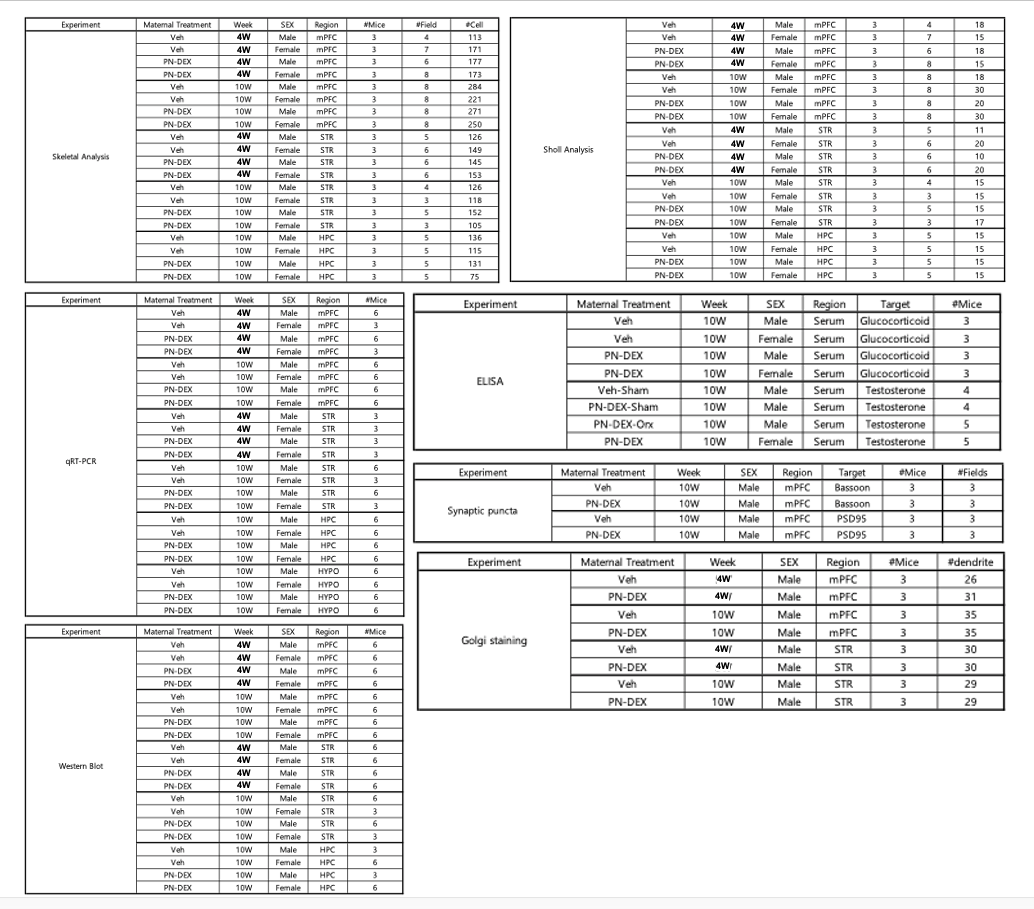
**

**Supplementary figure**

**
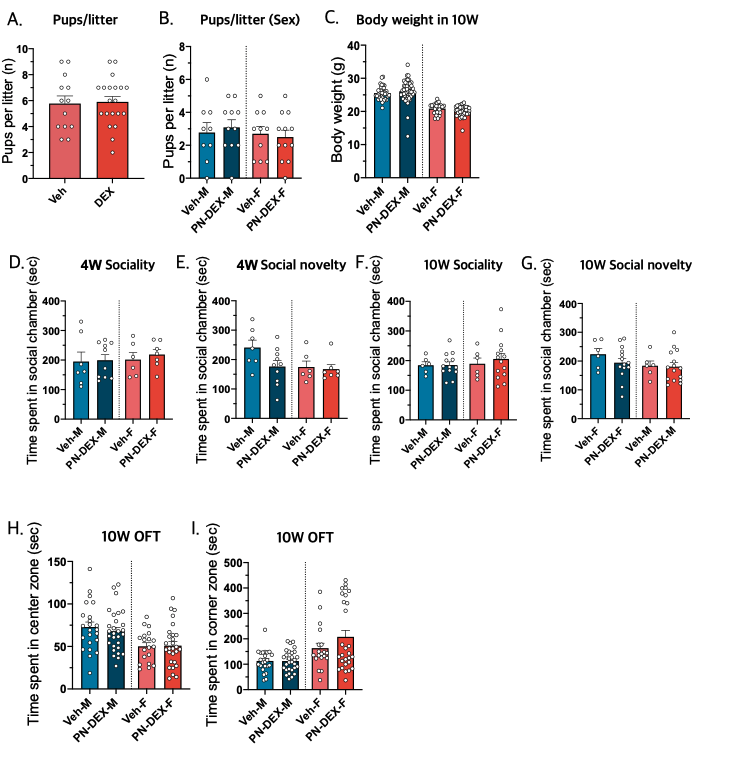
**

**Supplementary figure 1. Physiological data and behavior assessment in 4-week (4 W) and 10-week (10 W) offspring with maternal DEX exposure.**

(A) Number of pups per litter was counted. (B) The number of pups per litter is not sex-biased. (C) Body weights of adult mice were measured. (D-E) Time spent in a social chamber where unfamiliar mice resided was measured to assess the social behavior of adolescent and (F-G) adult mice. (H-I) The time spent in the center and corner zones was measured. Data are expressed as mean ± standard error of the mean (SEM). An unpaired t-test was conducted to determine differences between the PN-DEX and Veh groups.


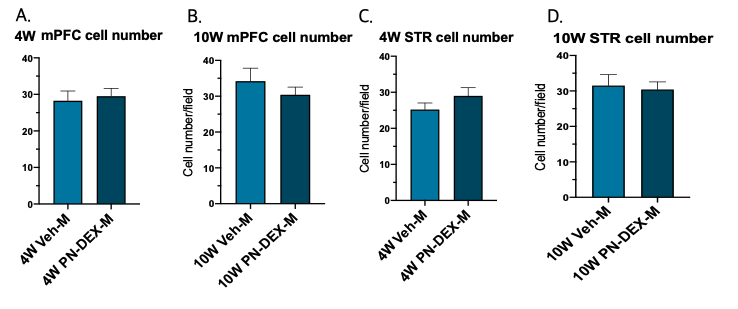


**Supplementary figure 2. The number of microglia in the mPFC and striatum in 4 W and 10 W mice.**

(A) Microglial cell number per field was counted in 4 W mPFC, (B) 10 W mPFC, (C) 4 W striatum, and (D) 10 W striatum. The data are expressed as mean ± standard error of the mean (SEM). An unpaired t-test was conducted to evaluate differences between PN-DEX and Veh.


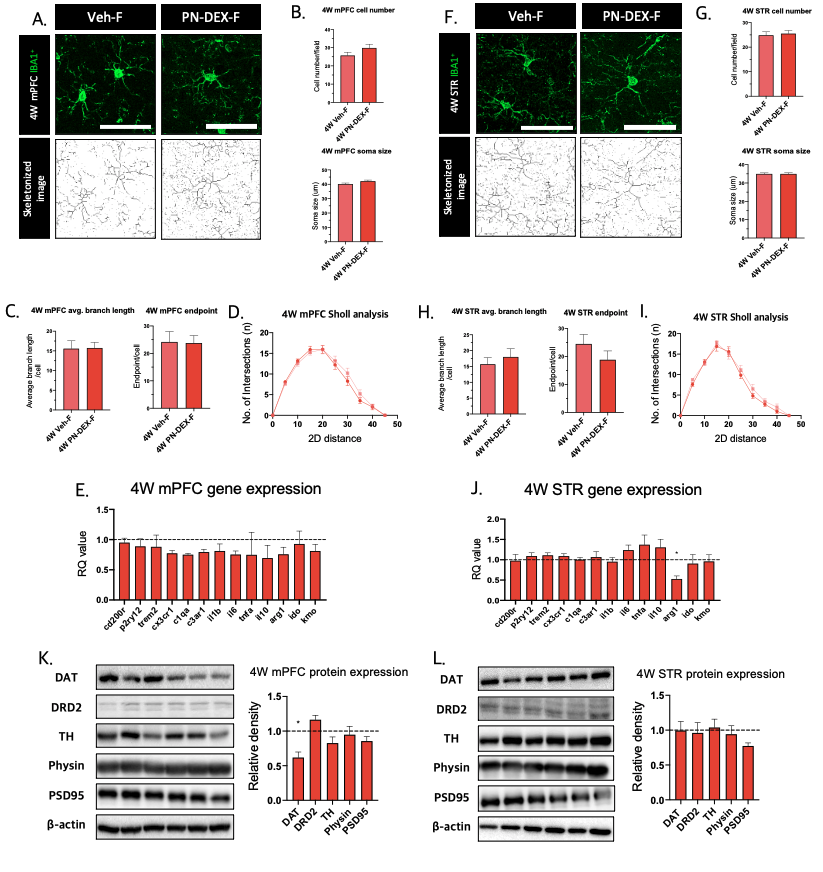


**Supplementary figure 3. No significant changes in microglia morphology, gene expression, and protein expression both in 4 W female mPFC and 4 W STR.**

(A) Representative images of Iba-1 positive microglial cells and their skeletonized images in the 4 W female mPFC (40x images, scale bar = 50 µm). (B) The number of microglia per field and soma sizes in the 4 W female mPFC were measured. (C) Branch length and the number of endpoints of microglial cells in the 4 W female mPFC were measured. (D) The number of microglial intersections in the 4 W female mPFC was analyzed. (E) qRT-PCR analysis used to determine the gene expression patterns in the 4 W PN-DEX-F mPFC compared to 4 W Veh-F. RQ values represent the ratio of respective genes as a percentage of Veh-F. (F) Representative images of Iba-1 positive microglial cell and their skeletonized images in a 4 W female STR (40x images, scale bar = 50 µm). (G) The number of microglia per field and the soma size in a 4 W female STR were measured. (H) Branch length and the number of endpoints of microglia cells in the 4 W female STR were measured. (I) The number of microglial intersections in the 4 W female STR was analyzed. (J) qRT-PCR analysis was used to determine the gene expression patterns in 4 W PN-DEX-F STR compared to that of 4 W Veh-F. The RQ values represent the ratio of respective genes as a percentage of Veh-F. (K) Dopamine-related and synaptic protein expression in the 4 W female mPFC and (L) 4 W female STR measured via western blotting. Relative density is the ratio of respective protein densities as a percentage of β-actin density.

Data are presented as mean ± standard error of the mean (SEM). An unpaired t-test was conducted to evaluate differences between the PN-DEX and Veh groups.


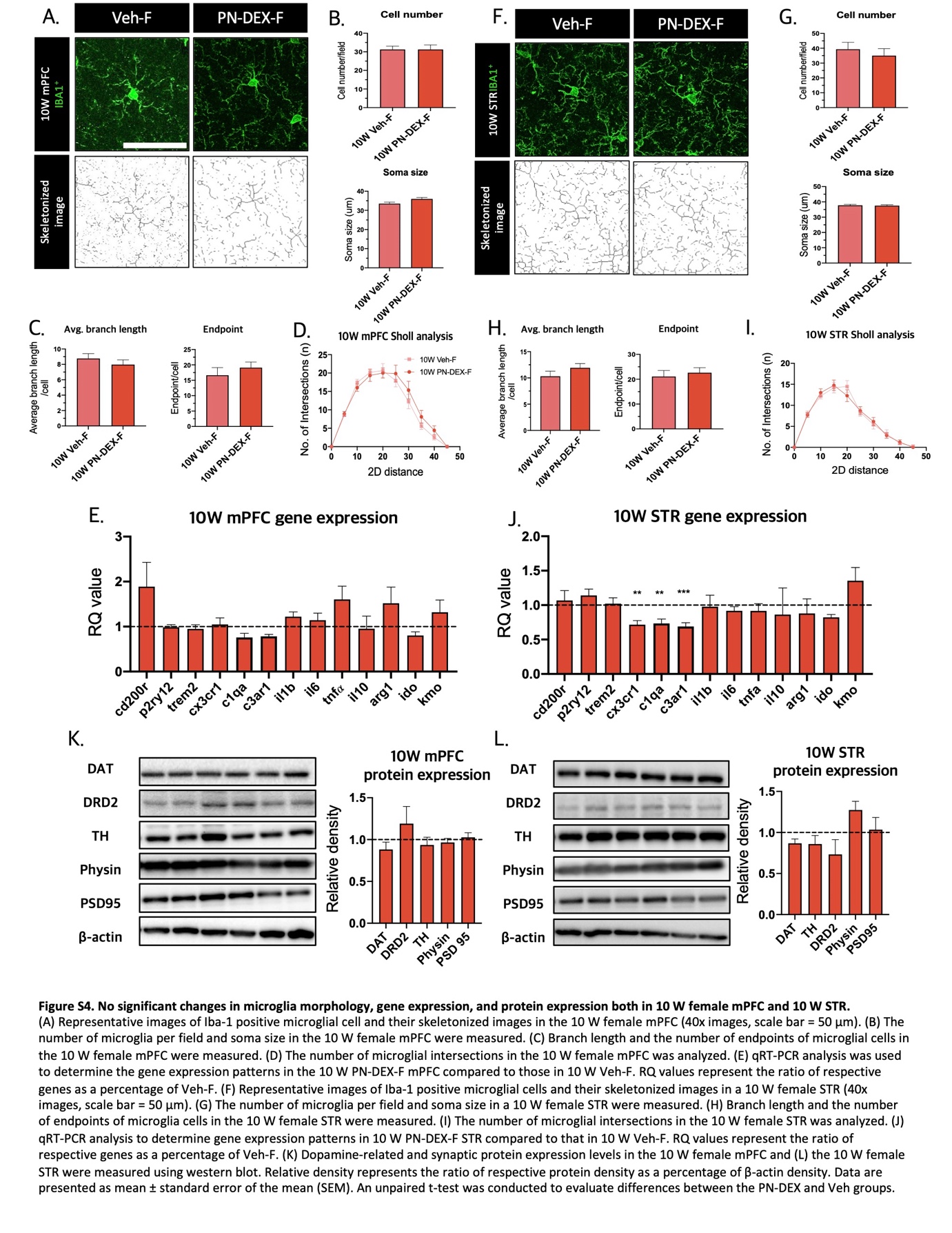


**Supplementary figure 4.** **No significant changes in microglia morphology, gene expression, and protein expression both in 10 W female mPFC and 10 W STR.**

(A) Representative images of Iba-1 positive microglial cell and their skeletonized images in the 10 W female mPFC (40x images, scale bar = 50 µm). (B) The number of microglia per field and soma size in the 10 W female mPFC were measured. (C) Branch length and the number of endpoints of microglial cells in the 10 W female mPFC were measured. (D) The number of microglial intersections in the 10 W female mPFC was analyzed. (E) qRT-PCR analysis was used to determine the gene expression patterns in the 10 W PN-DEX-F mPFC compared to those in 10 W Veh-F. RQ values represent the ratio of respective genes as a percentage of Veh-F. (F) Representative images of Iba-1 positive microglial cells and their skeletonized images in a 10 W female STR (40x images, scale bar = 50 µm). (G) The number of microglia per field and soma size in a 10 W female STR were measured. (H) Branch length and the number of endpoints of microglia cells in the 10 W female STR were measured. (I) The number of microglial intersections in the 10 W female STR was analyzed. (J) qRT-PCR analysis to determine gene expression patterns in 10 W PN-DEX-F STR compared to that in 10 W Veh-F. RQ values represent the ratio of respective genes as a percentage of Veh-F. (K) Dopamine-related and synaptic protein expression levels in the 10 W female mPFC and (L) the 10 W female STR were measured using western blot. Relative density represents the ratio of respective protein density as a percentage of β-actin density. Data are presented as mean ± standard error of the mean (SEM). An unpaired t-test was conducted to evaluate differences between the PN-DEX and Veh groups.


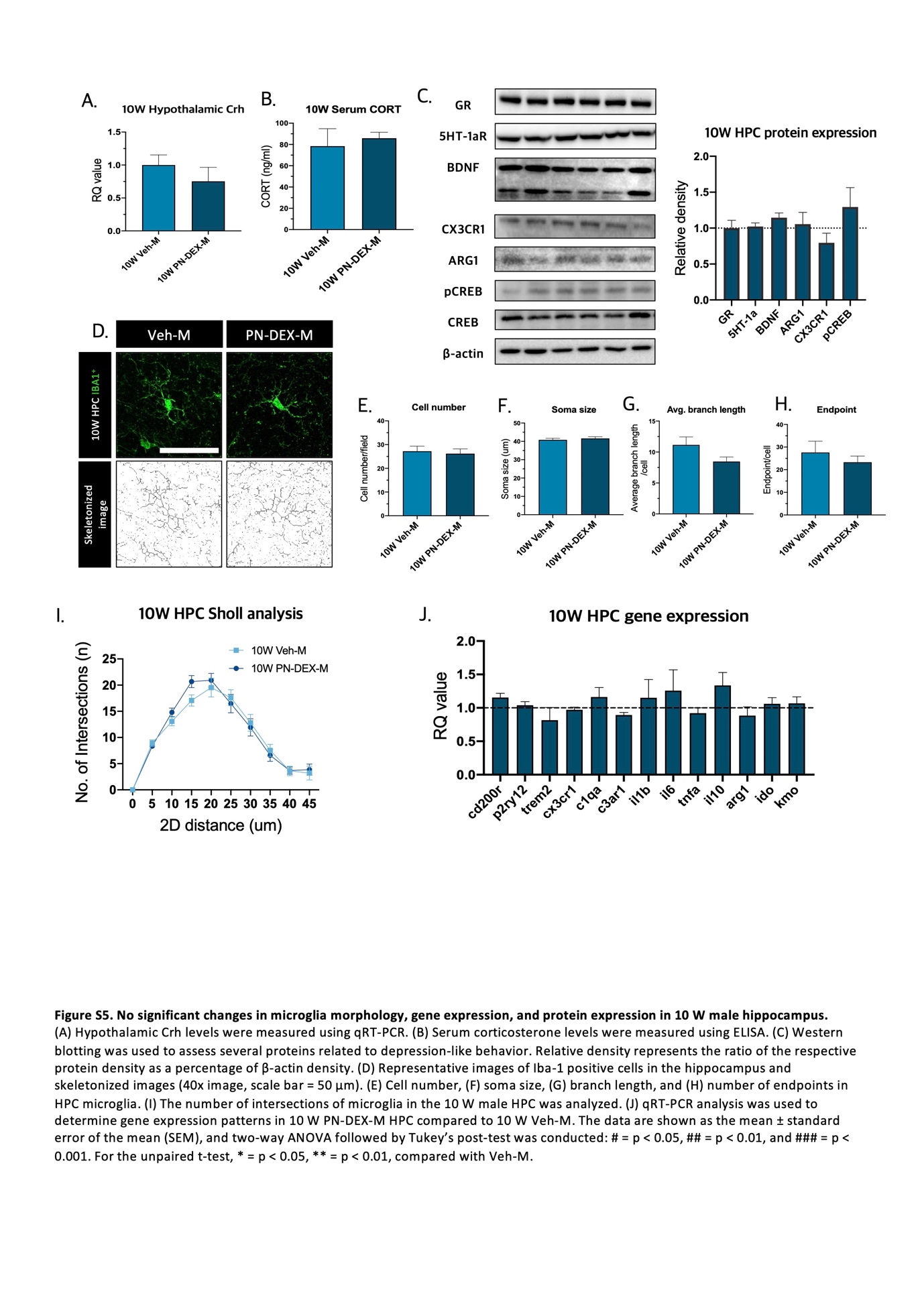


**Supplementary figure 5.** **No significant changes in microglia morphology, gene expression, and protein expression in 10 W male hippocampus.**

(A) Hypothalamic Crh levels were measured using qRT-PCR. (B) Serum corticosterone levels were measured using ELISA. (C) Western blotting was used to assess several proteins related to depression-like behavior. Relative density represents the ratio of the respective protein density as a percentage of β-actin density. (D) Representative images of Iba-1 positive cells in CA3 region the hippocampus and skeletonized images (40x image, scale bar = 50 µm). (E) Cell number, (F) soma size, (G) branch length, and (H) number of endpoints in HPC microglia (CA3 and dentate gyrus). (I) The number of intersections of microglia in the 10 W male HPC (CA3 and dentate gyrus) was analyzed. (J) qRT-PCR analysis was used to determine gene expression patterns in 10 W PN-DEX-M HPC compared to 10 W Veh-M. The data are shown as the mean ± standard error of the mean (SEM), and two-way ANOVA followed by Tukey’s post-test was conducted: # = p < 0.05, ## = p < 0.01, and ### = p < 0.001. For the unpaired t-test, * = p < 0.05, ** = p < 0.01, compared with Veh-M.
